# *Candida* Species Biofilms’ Antifungal Resistance

**DOI:** 10.3390/jof3010008

**Published:** 2017-02-21

**Authors:** Sónia Silva, Célia F. Rodrigues, Daniela Araújo, Maria Elisa Rodrigues, Mariana Henriques

**Affiliations:** Centre of Biological Engineering (CEB), University of Minho, Campus de Gualtar, 4710-057 Braga, Portugal; c.fortunae@gmail.com (C.F.R.); daniela.araujo@ceb.uminho.pt (D.A.); elisarodrigues@deb.uminho.pt (M.E.R.); mcrh@deb.uminho.pt (M.H.)

**Keywords:** candidiasis, biofilm, resistance, mechanisms

## Abstract

*Candida* infections (candidiasis) are the most prevalent opportunistic fungal infection on humans and, as such, a major public health problem. In recent decades, candidiasis has been associated to *Candida* species other than *Candida albicans*. Moreover, biofilms have been considered the most prevalent growth form of *Candida* cells and a strong causative agent of the intensification of antifungal resistance. As yet, no specific resistance factor has been identified as the sole responsible for the increased recalcitrance to antifungal agents exhibited by biofilms. Instead, biofilm antifungal resistance is a complex multifactorial phenomenon, which still remains to be fully elucidated and understood. The different mechanisms, which may be responsible for the intrinsic resistance of *Candida* species biofilms, include the high density of cells within the biofilm, the growth and nutrient limitation, the effects of the biofilm matrix, the presence of persister cells, the antifungal resistance gene expression and the increase of sterols on the membrane of biofilm cells. Thus, this review intends to provide information on the recent advances about *Candida* species biofilm antifungal resistance and its implication on intensification of the candidiasis.

## 1. Introduction

During the last two decades, the occurrence of *Candida* species infections has been increasing and becoming more difficult to treat due to the growth of immunogenic diseases, the disproportionate use of immunosuppressive drugs, malnutrition, endocrine disorders, the widespread use of indwelling medical devices, broad spectrum antibiotics, aging and an increase in patient’s population [1,2]. A fairly small number of *Candida* species are pathogenic for humans, causing superficial and deep-seated mycoses, disseminated worldwide [3]. Nonetheless, *Candida* is becoming a significant clinical problem that has taken the opportunity to create infections, called candidiasis [1,2,3].

*Candida albicans* continues to be the most prevalent and problematic of all *Candida* species. However, with the development of molecular identification methods, the number of other *Candida* species, non-*Candida albicans Candida* (NCAC) species, identified as implicated in candidiasis, is now superior. This group includes, among others, *Candida glabrata*, *Candida tropicalis*, *Candida parapsilosis* and *Candida krusei* species [4,5,6]. The pathogenicity of *Candida* species is attributed to certain virulence factors, such as the ability to evade host defences, adhesion and biofilm formation (on host tissues and or on medical devices), and the production of tissue-damaging hydrolytic enzymes, such as proteases, phospholipases and hemolysins [7]. Biofilms are biological communities with an extraordinary degree of organization, in which *Candida* cells form structured, coordinated, and functional communities, embedded in a self-secreted extracellular matrix. Biofilm production is also related to a high level of antifungal resistance of the associated microorganisms. Moreover, the ability of *Candida* species to form drug-resistant biofilms is an important factor in their contribution to human disease [8]. In the widely held view of *Candida* microbial biofilms [9] sessile cells within biofilms are less susceptible to antifungal agents than planktonic cells [10], since the development of drug resistance has been linked with, for example, an increase in the biofilm maturation process. 

Thus, the morbidity and mortality associated with candidiasis is still very high, even using the actual antifungal drugs [8,11]. Annually, 50% of adults and up to 30% of children [11,12] die of candidiasis, most of them related with biofilms. Furthermore, in fact, an estimated 100,000 deaths are caused by biofilm infections and about $6.5 billion are spent per year in the United States (US) in order to treat them. In fact, biofilm infections have been assumed as a serious public health problem with a high economic impact [13,14]. Thus, this review intends to provide information on the recent advances about *Candida* species biofilm antifungal resistance mechanisms and its implication on intensification of the candidiasis.

## 2. Candida Biofilms: A Real Problem

### 2.1. Biofilm Characteristics

The first documented scientific report about a biofilm was written in 1683 by Antoni van Leeuwenhoek in the Royal Society of London [15,16]. Since then, the definition of biofilm has evolved to what is now described as a community of adherent cells enclosed in an exopolysaccharide matrix, with properties distinct from those of free-floating (planktonic) cells [14,17,18,19]. Biofilms are often found attached to solid surfaces, but they can also be formed in liquid–air interfaces. The most common environments colonized by biofilms include aquatic environments, artificial structures, biomaterials, and plant and mammalian tissues. Within these environments, biofilms may be composed of a single-species population or a community derived from multiple species [6,17]. In both cases, biofilms are thought to provide ecologic advantages such as protection from the environment, nutrient availability, metabolic cooperation, and acquisition of new traits [19,20]. Perhaps because of these, biofilms are notoriously difficult to eliminate and are a source of many recalcitrant infections [6]. As such, biofilms are highly relevant to public health. Indeed, the National Institutes of Health signposts that pathogenic biofilms are responsible, directly or indirectly, for over 80% of all microbial infections [13,19,21], which can range from superficial mucosal (75% of women experience a vaginal yeast infection at least once in their lifetime) or dermal infections, to more serious spread infections with high mortality rates (47% in various cases) [13,21].

*Candida* species biofilms are among the most common in clinical settings, and their main characteristics, which are dependent of the *Candida* species, can be found in Table 1. *Candida* commonly adheres to biomedical devices, growing as a resilient biofilm capable of withstanding extraordinarily high antifungal concentrations [20,22]. The medical device most commonly infected by *Candida* biofilms is the central venous catheter (CVC), which is used to administer fluids and nutrients and/or cytotoxic drugs. The infusion fluid itself, or the catheter core, can be contaminated but, more frequently, *Candida* can have origin on the patient’s skin or on the hands of nursing staff (the distal tip of the catheter can be contaminated at the time of insertion or, instead, organisms can migrate down the catheter wound) [11,22,23,24]. Each year, in the US, more than five million CVCs are placed and, even with new improved clinical security procedures, biofilm infection still occurs in over 50% of these catheters [21]. Recurrently encountered and sometimes difficult to eradicate, superficial *Candida* infections related with medical devices are much less serious. The most common cases are those related with oral mucosa, e.g., denture stomatitis and silicone rubber voice prostheses [4,25,26]. Non-medical-device-related infections such as *Candida* endocarditis can result from the formation of biofilms on damaged vascular endothelium of native heart valves in patients with pre-existing cardiac disease [21]. Several authors reported formation of *Candida* biofilms on different surfaces, which are summarized in Table 2.

### 2.2. Candida Biofilms and Resistance Mechanisms

The major classes of antifungal drugs used for treatment of *Candida* species infections are azoles, polyenes, and echinocandins [86,104,105,106,107,108]. Azoles (e.g., fluconazole, voriconazole and posaconazole) possess a fungistatic effect, blocking ergosterol synthesis, targeting the enzyme lanosterol 14α-demethylase (related to the *ERG11* gene) and leading to an accumulation of toxic sterol pathway intermediates. Polyenes (e.g., amphotericin B and nystatin) are fungicidal, intercalating into membranes containing ergosterol, creating pores that destroy the proton gradient, which result in the outflow of the cytoplasm and other cell contents. Echinocandins (e.g., caspofungin, micafungin and anidulafungin) are also a fungicidal, targeting the synthesis of 1,3-β-glucan, a component of the *Candida* species cell wall. It is also important to address that preferably to azoles, the use of echinocandins and polyenes is recommended if the patient had prior azoles exposure and if the infection is markedly severe for patients infected with *C. glabrata*, which is consider as generally very azole-resistant. Echinocandins are, most frequently and according to the latest guidelines, the first antifungal drug choice in these severe cases of candidemia [109,110]. There is some evidence that suggests that prophylactic use of fluconazole may be advantageous for preterm neonates, transplant recipients, intensive care unit patients, and other high-risk patient populations [111,112,113,114,115,116]. Though, due to some controversies, this is not a standard for all hospitals [21].

**Figure 1:**
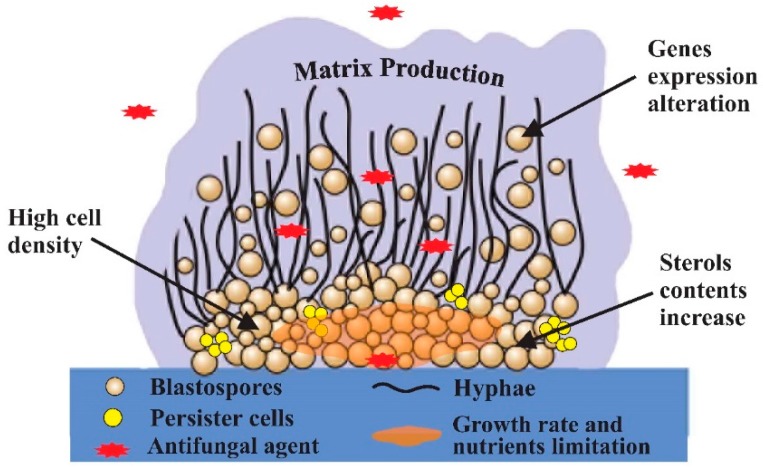
General scheme of the mechanisms described as involved on *Candida species* biofilm resistance.

Initial studies examined the impact of known mechanisms to play a role in drug resistance during planktonic *Candida* cells growth [7,117,118,119]. As described, acquired planktonic cell resistance has been linked to increased efflux pump activity, mutations in genes encoding drug target enzymes and alterations in the composition of both the cell membrane and the cell wall [117]. The *Candida* biofilm resistance phenomenon was for the first time demonstrated in 1995 for *C. albicans* by Hawser and Douglas (1995) [6]. After that, the ability of *Candida* species biofilms to survive extraordinarily to high antifungal concentrations has been the subject of numerous investigations for many researchers [7,117,118,120,121]. So, in the last decade, additional investigations began to focus on the role of biofilm-specific traits. These studies have examined the influence of high cell density, growth rate reduction, nutrient limitation, matrix extracellular production, presence of persister cells, gene expression alterations and sterols content increase on *Candida* membrane cells. The role of these mandatory factors is reviewed below and is schematized on Figure 1. 

#### 2.2.1. Impact of *Candida* Cells Density, Nutrient and Growth Limitation

An important biofilm-specific trait suspected to influence antifungal resistance is the high relative concentration of *Candida* cells into biofilm communities comparatively to the great majority of planktonic conditions [122,123]. Perumal and Chaffin, (2007) [122] after studying the cells density effect on antifungal treatment, observed that the azole antifungals’ tolerance to planktonic cell cultures was effectively lower when compared to intact and/or disrupt biofilm communities. *Candida albicans* biofilm formation is associated with the dimorphic switch between yeast and hyphal growth, and biofilms of this species generically have two distinct layers: a thin, basal yeast layer and a thickener compact hyphal layer [4]. In contrast, *C. parapsilosis* biofilms tend to be thinner, less structured, and consist almost exclusively of aggregates [29,124]. *Candida tropicalis* biofilms consist of a dense network of yeast cells with evident different filamentous morphologies and *C. glabrata* biofilms are structured on multilayers of blastospores with high cohesion among them [124]. In general, *C. glabrata* biofilms possessed higher density of cells comparatively to *C. tropicalis* and *C. parapsilosis* biofilms [125], which may be implicated on the usual highest resistance of *C. glabrata* biofilms to antifungal azoles and/or amphotericin B [7,117,120].

The well-structured biofilms layers open another hypothesis for *Candida* species antifungal resistance, that is, that cells placed in deeper layers of the biofilm grow slower owing to a lack of nutrients, and are subsequently more resistant to antifungal drugs. There is in fact a lack of work concerning this subject. However, by controlling nutrients in a perused biofilm fermentor, Baillie and Douglas [126,127] were able to compare the antifungal susceptibility of *C. albicans* biofilms growing at various rates. These authors, in opposition to what was expectable over a wide range of growth rates, verified that biofilm-associated cells exhibited similar levels of resistance to amphotericin B, suggesting that growth rate does not play a significant role in biofilm antifungal resistance. Similarly, *C. albicans* grown under glucose and iron limitation conditions were shown to both be highly resistant to amphotericin B [127]. Nevertheless, factors including pH, temperature, and oxygen availability are described as possible inductors of biofilm architecture alteration and thus the antifungal sensibility [128,129,130].

The general physiological state of sessile cells has also been reported as implicated in the susceptibility profiles of *Candida* biofilms. Metabolic activity confirms that cells within biofilms are undergoing mitochondrial respiration during development [5,29,86,123,130].

#### 2.2.2. Contribution of the Extracellular Matrix Production

Extracellular matrix (ECM) is a defining characteristic of all *Candida* species biofilms, providing the cells protection from hostile factors such as host immunity and antifungal agents [7,131]. In some of the pioneer works, *Candida* species biofilm’s matrices were shown to increase when biofilms were grown under dynamic flow conditions and their quantity is strongly species- and strains-dependent [6,124,132]. Subsequent work has shown that while ECM hampers diffusion, penetration of antifungal drugs is not thought to play an important role in biofilm resistance [132]. However, more recent studies have provided new insights about the chemical composition of ECM and that it may play a central role in resistance by antifungal agents’ neutralization. 

It is important to address that the composition of the *Candida* biofilm matrices is species-variable. Little is known about matrix composition of NCAC species biofilms, but according to Baillie and Douglas 1998 [126], *C. albicans* biofilm matrix is mainly composed of carbohydrates, proteins, phosphorus and hexosamines. Silva and colleagues 2009 [124] reported that the ECM of *C. parapsilosis* contained large amounts of carbohydrates and low levels of proteins. In the same study, *C. glabrata* biofilm matrices were found to have high levels of both proteins and carbohydrates, while *C. tropicalis* biofilm matrices had low levels of carbohydrates and proteins compared to the other NCAC species. Recently, Rodrigues et al. (2016) [120] revealed for the first time the presence of β-glucans in the *C. glabrata* matrices even when treated with amphotericin B. Furthermore, other authors [7] showed that matrix material extracted from biofilms of *C. tropicalis* and *C. albicans* contained carbohydrates, proteins, hexosamine, phosphorus and uronic acid. However, the major component quantified in *C. tropicalis* biofilm matrices was hexosamine (27%). The same authors also reported that these biofilms partially detached after treatment with lipase type VII and chitinase, which is in contrast to biofilms of *C. albicans* that detached only after treatment with proteinase K, chitinase, DNase I or β-*N*-aceytyglucosamidase. In *Candida* species, there is scarce knowledge concerning the contribution of extracellular DNA to biofilm matrix and overall structure [74].

In this sense, studies have been carried out to clarify the involvement of some of the matrix components in *Candida* biofilm resistance. Martins et al. (2013) [133] highlighted the importance of DNA in *C. albicans* biofilm formation, integrity and structure and that the addition of DNase improves the efficacy of polyenes and echinocandins, but not to azoles [133].

The major carbohydrate component is β-1,3 glucans, as treatment of *C. albicans* biofilms with β-1,3 glucanase helps detach biofilms from a substrate [132]. Its contribution is confirmed, where it was shown to increase in *C. albicans* biofilm cell walls compared to planktonic organisms and was also detected in the surrounding biofilm milieu and as part of the ECM [134]. The involvement in the resistance was realized when it was also shown that biofilm cell walls bound four- to five-fold more azole than equivalent planktonic cells, and culture supernatant bound a quantifiable amount of this antifungal agent. Moreover, β-1,3 glucanase markedly improved the activity of both fluconazole and amphotericin B. The addition of exogenous biofilm ECM and commercial β-1,3 glucan also reduced the activity of fluconazole against planktonic *C. albicans* in vitro [134]. The group has recently shown that the ECM β-1,3 glucan is synthesized from Fks1 using a defined knockout and over-expressing strain [135]. This study demonstrated that β-1,3 glucan is responsible for sequestering azoles, acting as a sponge and conferring resistance on *C. albicans* biofilms [135]. Further studies have shown that they are also responsible for sequestering echinocandins, pyrimidines, and polyenes [136]. Subsequent studies have identified a role for the *SMI1* in *C. albicans*, a gene involved in cell-wall glucans, in biofilm ECM production and development of a drug-resistant phenotype, which appears to act through transcription factor Rlmp and glucan synthase Fks1. In addition to *Fks1*, a zinc-response transcription factor *ZAP1* has been shown to be a negative regulator of ECM soluble β-1,3 glucan in both in vitro and in vivo *C. albicans* biofilm models [137]. Conversely, two glucoamylases, *Gca1* and *Gca2*, are thought to have positive roles in matrix production. A group of alcohol dehydrogenases *ADH5*, *CSH1*, and *LFD6* also have roles in matrix production, with *ADH5* acting positively, and *CSH1* and *LFD6* acting negatively [138]. It is also present on a number of other *Candida* species, including *C. glabrata*, *C. parapsilosis* and *C. tropicalis* [7].

Recent studies revealed the involvement of the matrix on *C. tropicalis* strains on amphotericin B resistance [118]. These studies highlight the incapacity of this traditional antifungal to totally prevent biofilm formation and to eradicate *C. tropicalis* biofilms. Interestingly, it was observed that amphotericin B led to a significant increase of the biofilm production due to an augment of the total protein and carbohydrate contents of the matrix. Fernandes et al. (2016) [121] revealed recently that voriconazole had no effect on pre-formed *C. tropicalis* biofilms. Remarkably, an increase in total biomass was observed when pre-formed biofilms were treated with this antifungal agent. This phenomenon is probably due to a response of *C. tropicalis* biofilm cells to the stress caused by the presence of the agent, which led to an expansion of the biofilm matrices’ production. Fonseca et al. (2014) [117] revealed a phenomenon similar for *C. glabrata* with an increase of proteins and carbohydrates in the matrices extracted from biofilms treated with fluconazole.

#### 2.2.3. Emergence of Persister Cells

An intriguing development in understanding *Candida* species biofilm resistance is the presence of persister cells [139]. Persister cells are a subset of cells, dormant variants, which lie deep in a biofilm and exhibit tolerance to multiple antifungal drug classes [140,141]. LaFleur (2006) [140] published the first study in fungi that demonstrated the presence of persister cells in *C. albicans* biofilms, but not in planktonic populations. In fact, a re-inoculation of cells, which survived from a biofilm treated with amphotericin B, was able to develop a new biofilm also with persister cells. This work suggested that these cells are not mutants but cells phenotype variants of the wild type. The presence of persisters in *C. krusei*, and *C. parapsilosis* biofilms treated with amphotericin B, were also described [142]. It was shown that the persister levels of the isolates varied from 0.2% to 9%, and strains isolated from patients with long-term carriage had high levels of persisters, whereas those from transient carriage did not [140]. While the mechanisms of *Candida* persister cells transition remains unclear, transcriptional analysis of these cells shows differential regulation of genes involved in ergosterol (*ERG1* and *ERG25*) and β-1,6 glucan (*SKN1* and *KRE1*) pathways [143]. Moreover, superoxide dismutases (SOD) were found to be differentially expressed by miconazole-treated sessile *C. albicans* cells compared to untreated cells. Inhibition of SOD resulted in an 18-fold reduction of the miconazole-tolerant persister cells and increased endogenous reactive oxygen species (ROS) levels in these cells [144]. Bink et al. (2011) [144] also demonstrated that in biofilms from strains lacking *sod4*/*sod5* at least three-fold less miconazole-tolerant persisters were observed, and ROS levels were also increased.

#### 2.2.4. Impact of Sterols Contents and Its Correlation with *ERG* Genes Expression

Ergosterol is the most prevalent sterol in *Candida* cells plasma membrane. Moreover, antifungal agents (e.g., azoles and amphotericin B) act as ergosterol synthesis inhibitors by binding to lanosterol demthylase, a specific enzymatic in ergosterol biosynthesis. The observation that *Candida* mutants with altered ergosterol synthesis show enhanced resistance to azoles and amphotericin B led the investigators to question if *Candida* biofilm cells may employ similar mechanisms of resistance [145]. Mukerjee and colleagues (2003) [145] showed that, when comparing planktonic cells’ membranes with the membranes of biofilm cells, the latter had a lower concentration of ergosterol, especially during the last steps of biofilm formation. This finding suggests that cells from mature biofilms rely less on ergosterol for maintaining its membrane fluidity and potentially limiting the efficacy of the ergosterol targeting drugs. In fact, several studies have demonstrated alterations in the transcriptional profile of sterol pathway genes in diverse *Candida* species [139,146]. *Candida albicans* microarray analysis demonstrated an increase of *ERG25* and *ERG11* in vitro biofilm growth when compared with its planktonic counterparts [147]. Interestingly, transcriptional analysis of a rat venous catheter biofilm also found increased transcription of *ERG25*, but not *ERG11* [134]. Moreover, the principal drug target, *ERG11*, can easily develop point mutations or even be over-expressed [148,149].

These results confirm the involvement of the alterations on sterol content in membrane of *C. tropicalis* cells as in other *Candida* cells. *Candida glabrata* is assumed to be the most azole-resistant species of all *Candida* species [7]. Besides, all the genes involved in the biosynthesis of ergosterol have been described as up-regulated in *C. glabrata* treated with azoles molecules in planktonic cells [7]. It is believed that the increase of *C. glabrata* infections is due to its intrinsically low susceptibility to azoles, including the imidazoles and the oral-parenteral triazoles (e.g., fluconazole, voriconazole) [150]. Additionally, it is known that the acquired resistance is resulted of rare mutations that are selected by drug pressure [151]. All the genes involved in the biosynthesis of ergosterol are expected to be up-regulated in the presence of azole molecules. Nevertheless, *ERG* genes are the ones more focused on in studies. Between them are *ERG1*, *ERG3*, *ERG6*, *ERG7*, *ERG9*, and especially *ERG11*. *ERG11* is noticeably more referred as the central point on the increase of ergosterol production, in response to the azole attack to the *C. glabrata* cell membrane, which has great ease to acquire azole resistance [151,152]. 

Induction of ergosterol genes has also been described in *C. dubliniensis*, where incubation with fluconazole and formation of biofilm was coupled with up-regulation of the *ERG3* and *ERG25* [153]. Moreover, up-regulation of genes involved with ergosterol biosynthesis has been described in *C. parapsilosis* biofilms [154], which are also resistant to azole antifungal therapy [155]. Regarding *C. tropicalis*, recently Fernandes and colleagues (2016) [121] demonstrated that, similar to *C. albicans,* voriconazole-resistant cells presented an increased on expression of *ERG* genes. 

#### 2.2.5. Over-Expression of Other Antifungal Targets

Many cases of drug resistance are linked to the increase of efflux pumps in *Candida* cells membrane and the consequent reduction of the drug accumulation within the cells [156]. In *C. albicans*, efflux pumps have been described as playing an important role in azole resistance, but not in resistance to amphotericin B and echinocandins [157,158,159,160,161]. 

The ATP binding cassette transporters (*CDR1* and *CDR2*) and major facilitator transporter (*MDR1*) are typically expressed at low levels in the absence of antifungal exposure [160]. The finding that azole-resistant clinical isolates often show constitutive over-expression of these pumps prompted investigators to postulate that the biofilm drug resistance phenotype may be related to increased efflux pump activity [159,162,163,164,165]. Ramage and colleagues (2009) [166] demonstrated that transcription of both *MDR1* and *CDR1* was more abundant in *C. albicans* biofilms than planktonic cultures of the same age. In opposition, some authors investigated the role of these efflux pumps by deletion of their genes and observed that, during the planktonic growth, these mutants displayed hypersensitivity to fluconazole. However, this phenotype was not observed when these same mutants were grown as biofilms, suggesting that the efflux pumps do not contribute significantly to drug resistance during the mature biofilm stage [131]. Mukherjee et al. (2011) [145] examined the role of efflux pumps in antifungal resistance throughout the biofilm process. The researchers included early, intermediate and mature *C. albicans* biofilms with planktonic growth comparisons. Similar to the prior investigation, single, double and triple mutants of the three main efflux genes were no more susceptible to fluconazole treatment during mature biofilm growth than the parent strains; however, in the early phase, double and triple efflux pump mutants had significantly increased azole susceptibility when compared with the parent strains. This suggests that the efflux pumps contribute to resistance during the early biofilm developmental phase, and that the pumps may function in a cooperative manner. This theory of time-specific efflux pump functionality was further supported by transcriptional analysis, showing higher expression of efflux pump genes after 12 h biofilm formation when compared with mature, 48 h biofilm formation [145]. This is collective evidence that *Candida* efflux pumps likely contribute to drug resistance during the early phase of biofilm growth, while their role in resistance in mature biofilms appears to be minimal at most. Investigations of *C. glabrata* and *C. tropicalis* biofilms have also shown up-regulation of efflux pumps [166,167]. Fonseca et al. (2016) [117] evaluated the expression of *C. glabrata ABC* (ATP-binding cassette) transporters (*CDR1*, *SNQ2* and *PDR1*) in presence of fluconazole, and observed that, in addition to high amounts, the matrix produced an over-expression of these efflux pumps.

This data supports the hypothesis that efflux pumps are an important—but not exclusive—determinant of fungal biofilm resistance to antifungal drugs. Their primary role may be for homeostasis within complex environments to protect themselves from acute toxicity, but within clinical environments, exposure to azoles drugs may enhance the levels of efflux pump expression, therefore either contributing towards or inducing clinical resistance. 

## 3. Conclusions

Reducing the incidence of biofilm-related candidiasis in hospitals is a requirement in the search for optimized patient care. However, the high degree of resistance of biofilm-associated *Candida* cells hinders rapid development toward highly efficacious therapies. Recent efforts of various excellent research groups tremendously broadened our knowledge on the complex mechanisms underlying biofilm resistance. According to the authors, the presence of matrix material is the most important biofilm resistance mechanism. However, several other important mechanisms such as cell density, differential regulation of drug targets, up-regulation of drug efflux pumps in developing biofilms, the presence of persisters into biofilms, up-regulation of different pathways and possibly yet-undefined mechanisms can further increase resistance to a maximum level. The elucidation of these resistance mechanisms provides a promising step toward the development of optimal therapies. 

## Figures and Tables

**Table 1:** Characteristics of the most common *Candida* species biofilms.

*Candida* Species	Biofilm Forming Capacity	Biofilm Characteristics	Refs.
*Candida albicans*	+++++	Basal blastospore layer with a dense overlying matrix composed of exopolysaccharides and hyphae.	[27,28]
*Candida dubliniensis*	++/+++	Chains of cells with thin extracellular matrix material.	[29,30]
		Hhigh variability among clinical isolates.	
*Candida glabrata*	++/+++	Forms considerably less biofilm than *C. albicans*.	[6,28]
		High in both protein and carbohydrate content.	
*Candida krusei*	++++	Thick multilayered biofilm of pseudohyphal forms embedded within the polymer matrix.	[31]
*Candida parapsilosis*	+++	Clumped blastospores and less volume.	[23,27,28,30,32]
		Large amounts of carbohydrate with less protein.	
		High variability among clinical isolates.	
*Candida tropicalis*	+++	Chains of cells with thin, but large, amounts of extracellular matrix material.	[24,28]
		Low amounts of carbohydrate and protein.	

+++ Normal to high; ++++ High; +++++ Very high biofilm formers.

**Table 2:** Localization, condition/disease and most common species found on several *Candida* biofilms.

Biofilm	Condition/Disease	Most Common *Candida* Species	Refs.
Medical Devices	Endocarditis	*Candida albicans*	[14,16,17,18,19,20,21,22,23,24,25,26,27,28,29,30,31,32,33]
	Total parenteral nutrition	*Candida glabrata*	
	Prosthetic joints	*Candida tropicalis*	
	Peritoneal dialysis	*Candida parapsilosis*	
	Cannulation		
	Ventriculoperitoneal shunts		
	Prosthetic knees		
	Hip joints		
	Breast implants		
	Bioprosthetic heart valves		
	Catheter-related disease: urinary catheter, central venous catheter, intravenous catheter		
Oral	Caries	*Candida albicans*	[10,24,33,34,35,36,37,38,39,40,41,42,43,44,45,46]
	Periodontal disease	*Candida glabrata*	
	Endodontic infection	*Candida dubliniensis Candida tropicalis*	
	Several mucosal infections	*Candida krusei*	
		*Candida parapsilosis*	
Gastrointestinal (GI) and Urinary Tract	Feeding tubes for enteral nutrition	*Candida albicans*	[47,48,49,50,51,52,53,54,55,56,57,58,59]
	Ulcerative colitis	*Candida tropicalis*	
	GI candidiasis		
	Pyelonephritis		
	Cystitis		
	Prostatitis		
	Intrauterine contraceptives		
Upper Airways	Rhinosinusitis	*Candida albicans*	[60,61,62,63,64,65,66,67,68,69,70,71,72,73,74,75,76,77,78,79,80,81,82,83,84,85]
	Ventilator-associated	*Candida glabrata*	
	Pneumonia	*Candida krusei*	
Lower Airways	Cystic Fibrosis	*Candida albicans*	[86,87,88,89,90,91,92,93,94,95,96,97,98,99]
	Allergic bronchopulmonary diseases		
Wounds	Diabetic foot ulcer	*Candida albicans*	[100,101,102,103]
	Non-healing surgical wounds	*Candida glabrata*	
	Chronic wound infections		
	Pressure ulcers		
	Venous leg ulcers		
